# A Meliorated Multi-Frequency Band Pyroelectric Sensor

**DOI:** 10.3390/s150716248

**Published:** 2015-07-06

**Authors:** Chun-Ching Hsiao, Sheng-Yi Liu, An-Shen Siao

**Affiliations:** 1 Department of Mechanical Design Engineering, National Formosa University, No. 64, Wunhua Rd., Huwei Township, Yunlin County 632, Taiwan; E-Mail: leouishen@gmail.com; 2 Department of Mechanical Engineering, National Taiwan University of Science and Technology, No. 43, Keelung Rd., Sec. 4, Taipei 10607, Taiwan; E-Mail: qbasic147@gmail.com

**Keywords:** pyroelectric sensor, multi-frequency, zinc oxide, aerosol deposition

## Abstract

This article proposes a meliorated multi-frequency band pyroelectric sensor for detecting subjects with various velocities, namely extending the sensing frequency under good performance from electrical signals. A tactic, gradually increasing thickness of the ZnO layers, is used for redeeming drawbacks of a thicker pyroelectric layer with a tardy response at a high-frequency band and a thinner pyroelectric layer with low voltage responsivity at a low-frequency band. The proposed sensor is built on a silicon substrate with a thermal isolation layer of a silicon nitride film, consisting of four pyroelectric layers with various thicknesses deposited by a sputtering or aerosol deposition (AD) method and top and bottom electrodes. The thinnest ZnO layer is deposited by sputtering, with a low thermal capacity and a rapid response shoulders a high-frequency sensing task, while the thicker ZnO layers are deposited by AD with a large thermal capacity and a tardy response shoulders a low-frequency sensing task. The fabricated device is effective in the range of 1 KHz~10 KHz with a rapid response and high voltage responsivity, while the ZnO layers with thicknesses of about 0.8 μm, 6 μm, 10 μm and 16 μm are used for fabricating the meliorated multi-frequency band pyroelectric sensor. The proposed sensor is successfully designed, analyzed, and fabricated in the present study, and can indeed extend the sensing range of the multi-frequency band.

## 1. Introduction

Pyroelectric sensors function by making use of the pyroelectric effect. Pyroelectricity can be described as the generation of surface charges during the change of spontaneous polarization with temperature. Materials with pyroelectric properties possess non-centrosymmetrical crystal structures which have a specific polar axis along the direction of the spontaneous polarization. The pyroelectric effect has been applied to environmental energy-harvesting systems. Pyroelectric energy conversion also offers a novel and direct way to convert time-dependent temperature fluctuations into electricity for micropower generators and low-energy-consumption systems [1,2,3]. A variable temperature in pyroelectric materials causes the generation of free charges on the surface, perpendicular to the direction of polarization. Pyroelectric devices are useful in many applications, such as pollution monitors, hot image detectors, intruder alarms, gas analysis, and temperature sensors. Thin-film pyroelectric sensors have many advantages, such as integration with on-chip circuitry, uncooled detection, room-temperature operation, speed, lower system costs, portability and a wide spectral response with high sensitivity [4,5,6,7]. A general pyroelectric sensor consists of a pyroelectric layer sandwiched between top and bottom electrodes, which is built on thermal-isolation structures or substrates for minimizing heat loss. The dynamic pyroelectric response current (*i_p_*) of the pyroelectric sensors is proportional to the absorption coefficient of radiation (*η*), the pyroelectric coefficient of the pyroelectric film (*P*), the electrode area (*A*) and the temperature variation rate of pyroelectric films (*dT/dt*). When a heat source with a constant temperature variation rate is applied on a pyroelectric device, the temperature variation rate in the pyroelectric device should be altered by changing the thickness of the pyroelectric film (thermal capacities), the structure of the pyroelectric film (trenches or cavities) and the material of the pyroelectric film (the absorption coefficient of radiation and the pyroelectric coefficient). The voltage responsivity (*R_v_*) can be expressed as [4]:

(1) $$R_{v}=\frac{R_{G}\eta PA\omega }{G_{T}\sqrt{\left(1+\omega^{2}\tau_{T}^{2}\right)}\sqrt{\left(1+\omega^{2}\tau_{E}^{2}\right)}}$$

where *R_G_* is the gate resistor, *τ_T_* is the thermal time constant (*τ_T_* = *H/G_T_*), *G_T_* is the thermal conductance to the surroundings, *H* is the thermal capacity (*H = c’ × d × A*)*, c’* is the volume-specific heat, *d* is the thickness of the pyroelectric element, *τ_E_* is the electrical time constant (*τ_E_* = *R_G_* × (*C_E_* + *C_A_*)), *C_E_* is the capacitance of the pyroelectric element, *C_A_* is the capacitance of the amplifier and ω is the frequency of the incident radiation. At a low frequency (*ω* << *τ_T_*^−1^), *R_v_* is proportional to the frequency and is shown as the following equation:

(2) $$R_{v}=\frac{R_{G}\eta PA\omega }{G_{T}}$$

Equation (2) can easily maximize *R_v_* by minimizing *G_T_* (*i.e.*, by adding a thermal insulation layer between the pyroelectric film and the substrates, adopting a suspended structure fabricated by a bulk micromachining technique using anisotropic silicon etching or by using a substrate with a low thermal conductivity). At a high frequency (*ω* >> *τ_T_*^−1^; *ω* >> *τ_E_*^−1^), *R_v_* is inversely proportional to the frequency and is shown as the following equation:

(3) $$R_{v}=\frac{\eta P}{c^{'}d(C_{E}+C_{A})\omega }$$

Equation (3) can easily maximize *R_v_* by minimizing the thermal capacity of the pyroelectric element *H* (*i.e.*, decreasing the thickness of the pyroelectric element). Hence, the frequency at *τ_T_*^−1^ is a watershed to distinguish the ranges of low and high frequencies, and the pyroelectric element’s thickness determines the value of the thermal time constant (*τ_T_* = *c’ × d × A/G_T_*) under the decided pyroelectric materials and electrode areas. Therefore, a thicker pyroelectric element increases the thermal time constant, which is suitable as the sensor for a low-frequency range. Unlike the thicker element, a thinner pyroelectric element reduces the thermal time constant, which is suitable as the sensor for a high-frequency range. It is difficult to apply a pyroelectric sensor with a single pyroelectric layer to multi-frequency sensing tasks when the materials and dimensions of the pyroelectric layers are already fixed. Although Hsiao *et al.* [5] were first to propose a novel multi-frequency band pyroelectric sensor, this sensor only used two pyroelectric films with various thicknesses. This led to the theory that the sensing frequency band was not fully expanded. Moreover, the redeeming effect of the voltage responsivity between the thicker and the thinner pyroelectric films was irregular and depended on the frequency. Therefore, in the present study, an improved structure consisting of four pyroelectric ZnO films with various thicknesses, top and bottom electrodes, a thermal isolation layer of a silicon nitride film and a silicon substrate for designing a multi-frequency band pyroelectric sensor was proposed. The four pyroelectric layers were mainly deposited by sputtering and aerosol deposition (AD). The thinnest pyroelectric layer was grown by sputter; the others were deposited by AD using a shadow mask method. The improved design further redeemed drawbacks of a thicker pyroelectric layer with a tardy response at a high-frequency band and a thinner pyroelectric layer with a low voltage responsivity at a low-frequency band by using the tactic of gradually increasing the thickness of the ZnO layers. Although more pyroelectric layers with various thicknesses were beneficial to the multi-frequency band pyroelectric sensor, the treatment of electrical signals with more pyroelectric layers was unrealistic.

Zinc oxide (ZnO) has a broad application ratio among the II-VI compound semiconductors. It also has a high ionicity compared with Si, Ge and III-V compounds. Besides, ZnO has a large exciton binding energy (about 60 meV), a wide band gap (3.37 eV), a non-stoichiometric defect structure, anisotropy in a crystal structure, strong absorption in the UV region, transparency in the visible region, a large variation in conductivity of high chemical stability, excellent thermal stability, low cost, low toxicity and great piezoelectric and pyroelectric potential. These properties make it useful in many applications, such as pyroelectric devices [7,8], gas sensors [9] and film bulk acoustic resonators [10]. The thinnest ZnO film was deposited by RF sputtering. Sputter deposition is a physical vapor deposition (PVD) method of depositing thin films by sputtering. Sputter deposited films have a composition close to that of the source material. The properties of ZnO films are affected by the sputtering conditions such as the composition of mixed process gases, working pressure, substrate temperature, radio frequency (RF) power, the gap between the target and substrate and the post-annealing temperature. Moreover, thicker ZnO film was grown by AD. In this technique, submicron ceramic particles were accelerated by the gas flow in the nozzle up to a velocity of several hundred meters per second and sprayed onto the substrate without vaporization of the materials. AD provides many advantages for producing films in the range of 1~100 μm in thickness with a high deposition rate, low deposition temperature, and low cost. The AD method can achieve fine patterning and fabricate a dense structure by the reduction of the crystallite’s size by fracture or plastic deformation at room temperature [11,12,13]. Although the ZnO film grown at a low temperature is available from the AD process, furnace annealing was adopted to improve the ZnO film quality by reducing defects in the present study. The furnace annealing parameters include temperature, duration, atmosphere and pressure. In particular, annealing temperature and duration are the most effective factors for improving film quality.

The responsivity is proportional to the temperature variation rates in the pyroelectric layers. In the present study, transient temperature fields in the meliorated multi-frequency band pyroelectric sensors were simulated to probe into temperature variation rates at various pyroelectric layers and estimate the response of the sensors. Then, an improved multi-frequency band pyroelectric sensor was accomplished by meliorating the sensor’s structure, analyzing the temperature variation fields at various pyroelectric layers, fabricating the meliorated multi-frequency band pyroelectric sensor by the MEMS process, measuring the responsivity of the sensors by a measurement system, and treating and integrating the electrical signals of the sensors using a LabVIEW system.

## 2. Materials and Methods

### 2.1. Improved Design for a Multi-Frequency Band Pyroelectric Sensor

**Figure 1:**
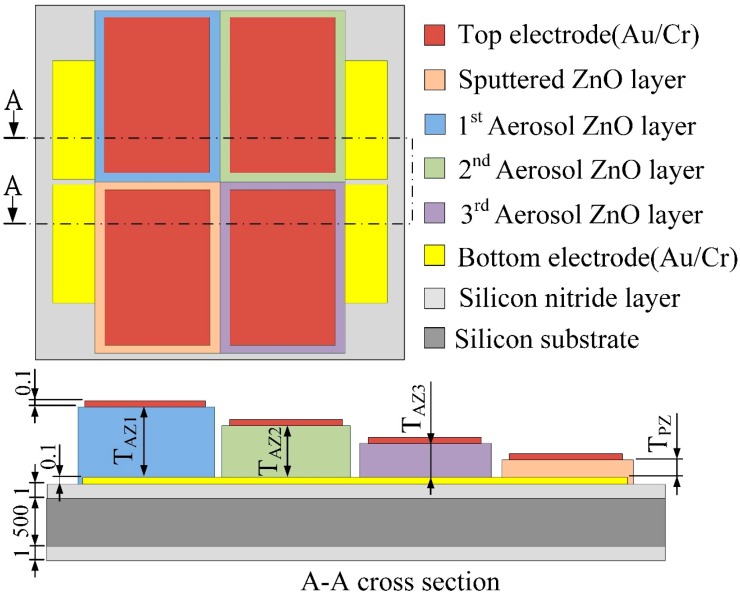
Schematic diagram of the meliorated multi-frequency band pyroelectric sensor (unit: μm).

A general pyroelectric sensor is composed of a single pyroelectric layer sandwiched between top and bottom electrodes and built on a substrate with an insulating layer. As the thicknesses of the pyroelectric layers determine the thermal time constant (*τ_T_*) under pyroelectric materials and electrode areas already fixed, the thermal time constant determines the optimal working frequency of the pyroelectric sensors. The meliorated multi-frequency band pyroelectric sensor, consisting of four ZnO pyroelectric layers with various thicknesses, and top and bottom electrodes, was built on a silicon substrate with a thermal-insulation (silicon nitride) layer to reduce heat and electric loss. Figure 1 shows the schematic diagram of the meliorated multi-frequency band pyroelectric sensor. The thinnest ZnO pyroelectric layer was deposited by sputtering with high qualities, and the others were deposited by AD. The sputtered ZnO layer acted as a producer of the responsivity at higher frequency bands, while the aerosol ZnO layers detected the electrical signals of the sensors at lower frequency bands. In the AD, the starting powder was commercially available ZnO (Top Nano Technology Co. Ltd., New Taipei, Taiwan). The properties of the starting ZnO powder are shown in Table 1. Table 2 shows the process parameters used for the AD method. The powder was subjected to heat treatment at 150 °C for 1 h using an oven to reduce moisture content and agglomerated particles in the powder before ZnO films were deposited. ZnO powder with high moisture content became severely agglomerated particles for absorbing the kinetic energy when the powder impacted against the substrate. Subsequently, furnace annealing was used on the ZnO films to improve film quality. A furnace annealing system (SJ High Technology Company, Taiwan), consisting of a single zone tube furnace with tube furnace Model T21-303, a single zone programmable temperature control console with controller Model SJ-C01, a cooling system, and a vacuum system, were used for the ZnO film annealing in an N_2_ ambient. The process parameters of furnace annealing for the ZnO films are shown in Table 3. The film thickness was further probed by a surface analyzer (ET-4000AK, Kosaka, Tokyo, Japan). The XRD patterns and the SEM micrographs of the ZnO layers could refer previous studies [7,14].

**Table 1:** Material properties and geometry of commercially prepared ZnO powder.

Items	Data
Appearance	White powder
Density	5.61 g/cm^3^
Specific surface area	17 m^2^/g
Particle form	Sheet
Particle size	300 nm (Diameter) × 20 nm (Thickness)

**Table 2:** Process parameters for ZnO films deposited by the AD method.

Items	Data
Starting powder	ZnO
Substrate	Silicon
Pressure difference between deposition and aerosol chambers	140 (Torr)
Carrier gas	Nitrogen
Consumption of carrier gas	3 (L/min)
Orifice size of nozzle	0.4 × 10 (mm × mm)
Substrate temperature	25 (°C)
Deposition area	70 × 70 (mm × mm)
Distance between nozzle and substrate	5 (mm)
Scanning rate	10 (mm/s)
Deposition rate	8.2 (nm/s)

**Table 3:** Process parameters for ZnO films treated by furnace annealing.

Items	Data
Holding temperature	800 (°C)
Duration	15 (min)
Speed for raising temperature	10.4 (°C/min)
Ambience	Nitrogen
Cooling type	Cooling in furnace

### 2.2. Simulation for Estimating Voltage Responsivity

The meliorated multi-frequency band pyroelectric sensor used both sputtering and AD to extract the advantages of the thinner and thicker ZnO pyroelectric films to further integrate the electrical outputs. Temperature variation rates in the pyroelectric layers markedly affected the responsivity of the pyroelectric sensors. The response is in proportion to the temperature variation rate in the pyroelectric layers. The dynamic pyroelectric response current of pyroelectric devices can be expressed as [4]:

*i_p_ = η × P × A × dT/dt* (4)

**Figure 2:**
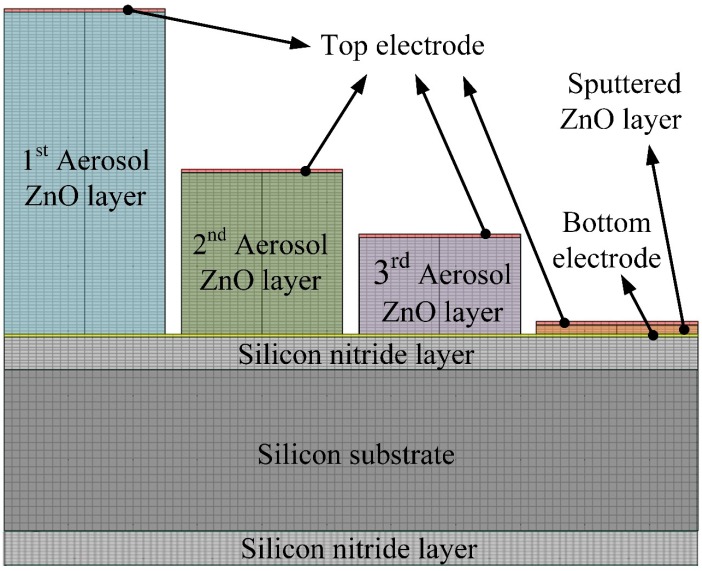
Two-dimensional numerical model for the multi-frequency band ZnO pyroelectric sensor.

That is, the higher the temperature variation rate in pyroelectric films, the higher the response of the pyroelectric sensors. However, the temperature variation field is difficult to extract from thin films by experimental measurements. Hsiao *et al.* [2,3,8] also used commercial multiphysics software, COMSOL MULTIPHYSICS^®^ (Stockholm, Sweden), to explore the temperature variation rates in ZnO pyroelectric devices in the designing and optimizing of a partially covered mesh-type electrode. In the present study, a two-dimensional finite element model was constructed using the commercial multiphysics software, COMSOL MULTIPHYSICS^®^ 4.2, to explore the temperature variation rate in meliorated multi-frequency band ZnO pyroelectric sensors. The materials’ properties of the films and substrate are shown in Table 4. There was an isotropic assumption for the films and substrate properties in this model. The model was meshed using a regular mesh, as shown in Figure 2. The thickness of the sputtered ZnO layer (T_PZ_) was fixed as 0.3 μm, while the thicknesses of the aerosol ZnO layers (T_AZ1_, T_AZ2_, T_AZ3_) were 3, 1 and 0.6 μm. The incident irradiation power applied on the top side of the meliorated multi-frequency band pyroelectric device was nearly 1.228 × 10^−12^ W/μm^2^ [15]. The thermal isolation condition was applied to the rear side of the silicon substrate, and the symmetric condition was applied to the two lateral sides as boundary conditions.

In fact, pyroelectric devices use voltage responsivity for presenting the performance of the sensors. Hence, voltage responsivities were calculated for estimating the electrical outputs of the sensors. The voltage responsivity (*R_v_*) is defined as the signal generated when pyroelectric sensors are exposed to a modulated radiation. Moreover, when pyroelectric sensors are connected to a high impedance amplifier, the observed signal is equal to the voltage produced by the charge. *R_v_* can be expressed as [4]:

(5) $$R_{v}=\frac{i_{p}}{Y\times W_{0}}$$

*i_p_* = η × P × A × dT/dt (6)

where *i_p_* is the dynamic pyroelectric response current, *W_0_* is the magnitude of the incident radiation and *Y* is the electrical admittance, as in:

(7) $$Y=R_{G}^{-1}+i\text{ω}C_{T}$$

where *R_G_* is the gate resistor, ω is the modulated frequency of the incident radiation, *C_T_* is the sum of *C_E_* and *C_A_*, *C_A_* is the capacitance of the amplifier, *C_E_* is equal to ε*_0_* × ε*_r_* × *A*/*d*, ε*_0_* is the vacuum permittivity (8.85 × 10^−12^ F/m) and ε*_r_* is the relative permittivity or dielectric constant of the materials. *R_v_* was estimated using the simulated data of the temperature variation rates and Equations (5)–(7).

**Table 4:** Material properties used for the simulation of temperature variation fields.

Materials	Thermal Conductivity (Wm^−1^·K^−1^)	Specific Heat (Jg^−1^·K^−1^)	Density (g·cm^−3^)	Thickness (μm)
Silicon substrate	163	0.703	2.330	5
Silicon nitride	20	0.700	3.100	1
Electrodes	317	0.129	19.300	0.1
Sputtered and aerosol ZnO layers	6	0.125	5.676	T_PZ_ (0.3μm)
				T_AZ1_ (3μm)
				T_AZ2_ (1μm)
				T_AZ3_ (0.6μm)

### 2.3. Fabrication Flow

**Figure 3:**
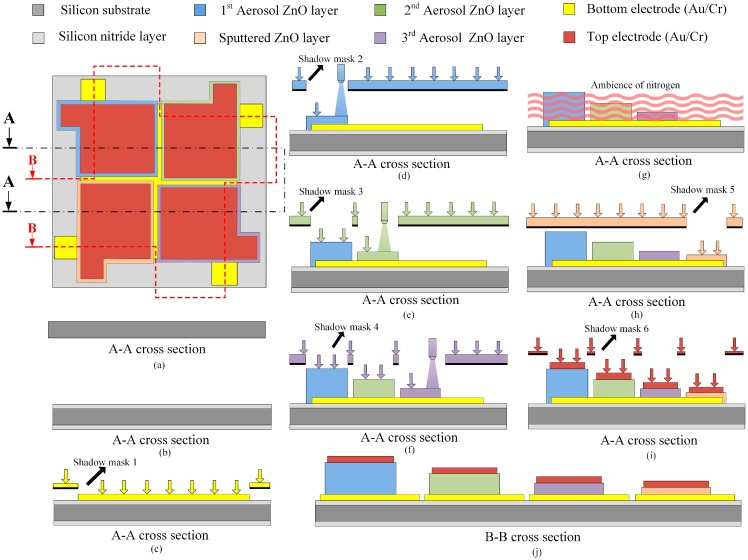
Fabrication procedure of the meliorated multi-frequency band pyroelectric sensor.

**Figure 4:**
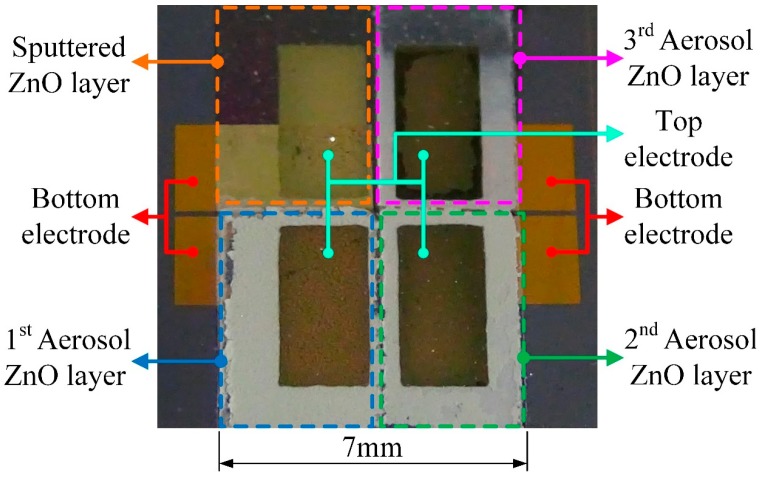
Fabricated multi-frequency band pyroelectric sensor with an improved design.

The fabrication flow of the meliorated multi-frequency band ZnO-film pyroelectric sensor was divided into ten steps, as follows. A silicon wafer with specifications of (100) p-type, double-side polished and resistivity of 1–10 Ω-cm, was used as a substrate to build the multi-frequency band pyroelectric sensor, as shown in Figure 3a. A 1 μm thicker low-stress silicon nitride layer was deposited on both sides of the substrate by LPCVD, which was mainly for reducing or blocking heat or electric loss through the silicon substrate, as shown in Figure 3b. Then, the bottom electrode consisting of a 100 nm-thick gold layer and a 10 nm-thick chromium layer, was deposited on the substrate by electron beam (E-beam) evaporation and patterned by the shadow mask method, as shown in Figure 3c. The shadow mask method can simplify process steps and shorten processing time. Chromium was an adhesion layer to promote the adhesion between the gold and the substrate. The next step was to deposit the aerosol ZnO films with three thicknesses of about 3, 1 and 0.6 μm by AD with three shadow masks, as shown in Figure 3d,f, and then, those were promoted by furnace annealing, as shown in Figure 3g. Subsequently, a ZnO target with 99.99% purity was adopted to deposit a 0.3 μm thick ZnO layer (the sputtered ZnO layer) onto the bottom electrode by RF magnetron sputtering (as shown in Figure 3h), which was pre-sputtered for 15 minutes to remove any surface impurities before the film was deposited. The chamber was pumped with a base pressure of up to 8 × 10^−7^ Torr before sputtering. The chamber was then filled with a mixture of argon and oxygen with a gas-mixing ratio of 5:3. The RF power was kept at 120 W, and the chamber pressure was 2 mTorr during the film deposition. The substrate was heated up to 200 °C while deposited, which could enhance the ZnO film quality. The top electrode was deposited on the ZnO films by an E-beam and patterned by the shadow mask method, as shown in Figure 3i. The composition of the top electrode was the same as that of the bottom electrode. Finally, a wet etchant of CH_3_COOH:H_3_PO_4_:H_2_O = 1:1:10 was adopted to pattern the ZnO layers to open the bonding pads of the bottom electrodes, as shown in Figure 3j. The meliorated multi-frequency band pyroelectric sensor was fabricated as shown in Figure 4.

### 2.4. Voltage Responsivity Treatment and Measurement

**Figure 5:**
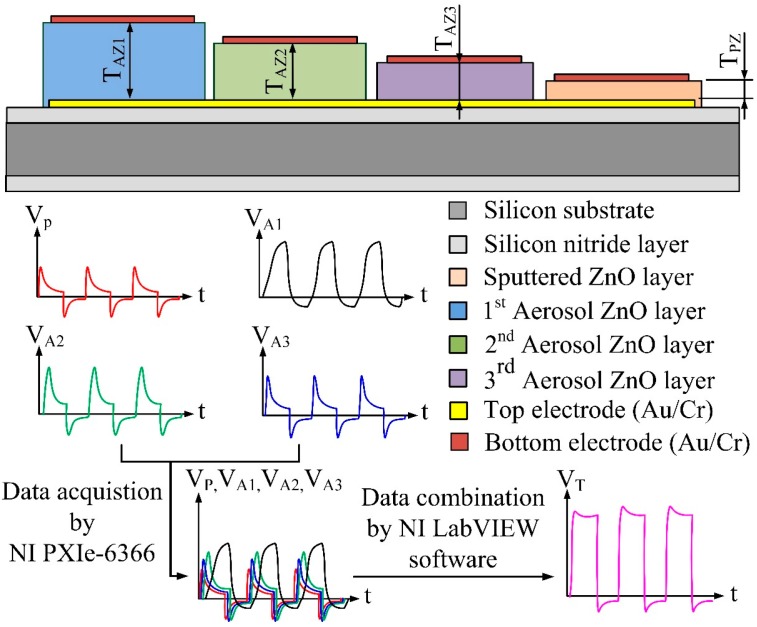
Schematic diagram for electrical signal treatment procedure.

**Figure 6:**
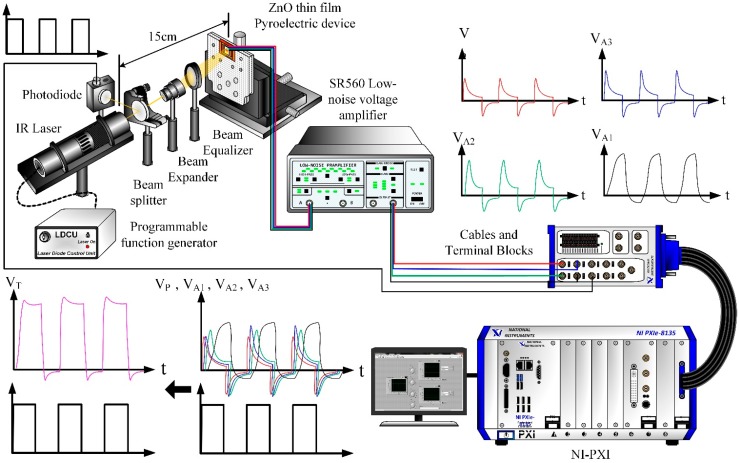
Schematic diagram for voltage responsivity measurement system.

The meliorated multi-frequency band pyroelectric sensor redeemed the drawbacks of the thinner and thicker ZnO pyroelectric films to integrate the electrical outputs generated from those films into an all-round signal. A schematic diagram of the signal treatment is shown in Figure 5. The voltage responsivity of V_P_ was generated from the sputtered ZnO film for shouldering a high-frequency response, and the voltage responsivities of V_A1_, V_A2_ and V_A3_ were generated from the aerosol ZnO films for taking a low-frequency response. The integrated and treated electrical signal was V_T_. A responsivity measurement system, as seen in Figure 6, was used to evaluate the performance of the meliorated multi-frequency band ZnO thin-film pyroelectric sensor. This system consisted of an infrared laser, a programmable function generator, a prism, a photodiode, a beam expander, a beam equalizer, a low-noise voltage amplifier and NI LabVIEW system. The radiation source was a calibrated infrared (IR) laser with 900 nm wavelength and 7 mW maximum power. The IR laser beam was molded as a square wave with a modulated frequency (ω) by a programmable function generator. A prism was used to split the modulated beam into two beams, which had the same power: one was reflected on a photodiode as the reference signal, and the other was expanded and equalized via a beam expander and a beam equalizer such that the beam spot could uniformly cover the entire region of the top electrode of the sensors. The responsivities of V_P_, V_A1_, V_A2_ and V_A3_ were filtered, amplified, modulated, and combined as an integrated voltage responsivity (V_T_) by NI LabVIEW software. Moreover, both the integrated voltage responsivity of the sensors and the reference signal of the photodiode were acquired, recorded and displayed using an NI LabVIEW system consisting of case NI PXIe-1082, controller NI PXIe-8135, data acquisition card NI PXIe-6366 and NI LabVIEW 2013 software.

## 3. Results and Discussion

**Figure 7:**
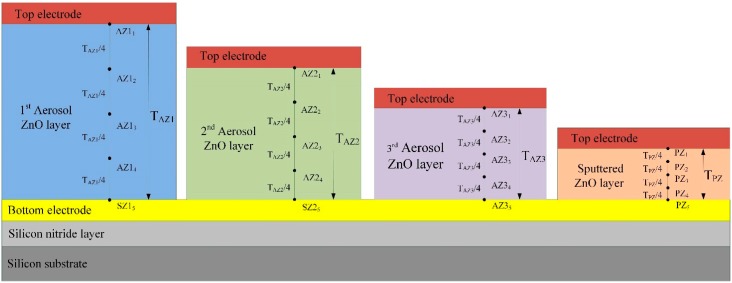
Points defined for explaining the transient temperature fields in the meliorated multi-frequency band pyroelectric sensor.

Our study clearly shows that a large and rapid temperature variation is a favorable condition for generating high electrical signals. Transient temperature fields in multilayer ZnO thin film pyroelectric sensors were simulated, and the response time of the sensors was estimated. Furthermore, the reduction of the pyroelectric element’s thickness leads to a reduced heat capacity and a raised temperature variation of the element. However, this increases the electrical capacity of the element and decreases the voltage responsivity of pyroelectric sensors. Therefore, the data regarding temperature variation rates and response times were further used to calculate the voltage responsivity of pyroelectric sensors. The points, as shown in Figure 7, were used to interpret the temperature variation rates in a meliorated multi-frequency band pyroelectric sensor. Points PZ_1_, PZ_3_ and PZ_5_ were, respectively, located on the top, the middle and the bottom of the sputtered ZnO layer. The thickness of the sputtered ZnO layer was named T_PZ_. Points AZ1_1_, AZ1_3_ and AZ1_5_ were, respectively, located on the top, the middle and the bottom of the first aerosol ZnO layer. The thickness of the first aerosol ZnO layer was named T_AZ1_. Similarly, points AZ2_1_, AZ2_3_ and AZ2_5_ were, respectively, located on the top, the middle, and the bottom of the second aerosol ZnO layer. The thickness of the secondaerosol ZnO layer was named T_AZ2_. Points AZ3_1_, AZ3_3_ and AZ3_5_ were, respectively, located on the top, the middle, and the bottom of the third aerosol ZnO layer. The thickness of the third aerosol ZnO layer was named T_AZ3_.

The temperature variation rate increased when the point approached the top side of the ZnO layer because incident radiation was applied on the top electrode. Using the temperature variation rate at the point nearest the top electrode did not discriminate the properties of the pyroelectric sensor with various thicknesses of ZnO films. Therefore, a conservative consideration was to adopt the properties at the point nearest the bottom electrode to inspect the sensors because the temperature variation rate at this point could display the difference in ZnO films with various thicknesses. Therefore, promotion in the temperature variation rate at the point near the bottom electrode possessing the lowest temperature variation rate could certainly enhance the responsivity of pyroelectric sensors.

**Figure 8:**
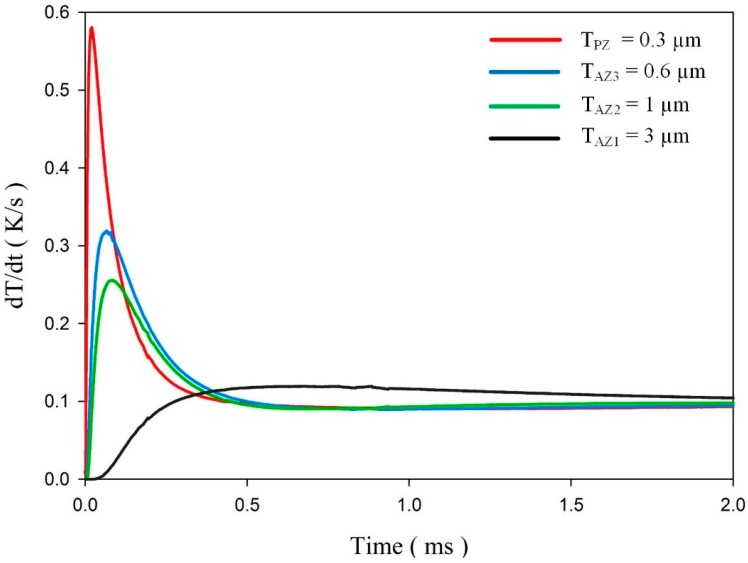
Relationship between the temperature variation rate and time at points PZ_5_, AZ1_5_, AZ2_5_ and AZ3_5_ in a meliorated multi-frequency band pyroelectric sensor under sputtered ZnO film with a constant thickness of 0.3 μm and aerosol ZnO films with three thicknesses from 0.6 to 3 μm.

For the meliorated multi-frequency band pyroelectric sensor, Figure 8 shows the relationship between the temperature variation rate and time at points PZ_5_, AZ1_5_, AZ2_5_ and AZ3_5_, when the sputtered ZnO film, with a constant thickness of 0.3 μm, and the aerosol ZnO films, with three thicknesses from 0.6 to 3 μm, were used to fabricate the ZnO pyroelectric sensors. The relative conditions for computing the voltage responsivity are shown in Table 5. A thinner ZnO film possessed a higher temperature variation rate and a shorter response time, while a thicker ZnO film possessed a lower temperature variation rate and a longer response time. Increasing the thickness of the ZnO layer reduced the temperature variation rate and increased the peak time of the maximum temperature variation rate. Therefore, a single ZnO film with a constant thickness in a pyroelectric device could not handle various multi-frequency sensing tasks.

**Table 5:** Relative conditions for calculating *Rv*.

R_G_ (MΩ)	*C_A_* (pF)	*P* (10^−4^ C/m^2^·K)	*A* (mm^2^)	*ε_r_* (Unit-Less)	*W_0_* (W/μm^2^)
22	6	0.1	9	11	1.228 × 10^−12^

**Figure 9:**
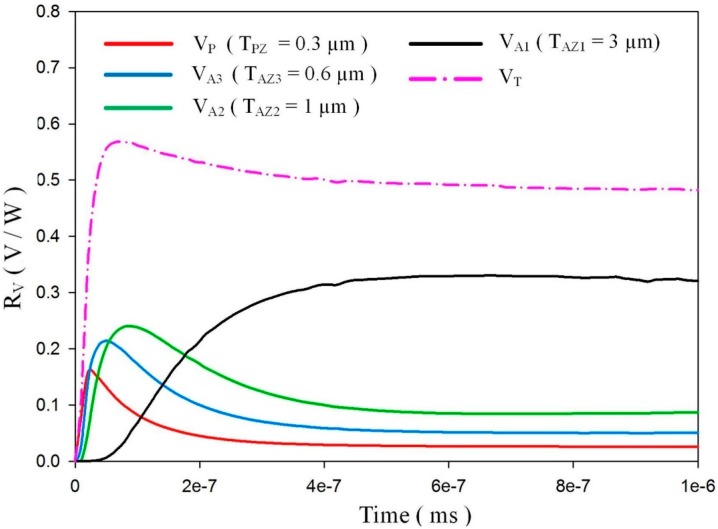
Relationship between the calculated voltage responsivities and time in a meliorated multi-frequency band pyroelectric sensor under sputtered ZnO film with a constant thickness of 0.3 μm and aerosol ZnO films with three thicknesses from 0.6 to 3 μm.

Figure 9 shows the relationship between the voltage responsivities and time in a meliorated multi-frequency band pyroelectric sensor when sputtered ZnO film with a constant thickness of 0.3 μm and aerosol ZnO films with three thicknesses from 0.6 to 3 μm were used to fabricate a meliorated multi-frequency band pyroelectric sensor. Furthermore, the temperature variation rates at points PZ_5_, AZ1_5_, AZ2_5_ and AZ3_5_ were used to estimate the voltage responsivities generated by the sputtered and aerosol ZnO films, respectively. The curve of the voltage responsivity generated by sputtered ZnO film (V_P_) with a rapid response could be attributed to a thinner ZnO layer with a large temperature variation rate and a small thermal capacity, while that generated by aerosol ZnO films (V_A1_, V_A2_ and V_A3_) with a tardy response could be attributed to a thicker ZnO layer with a large thermal capacity and a small temperature variation rate. Hence, the V_P_ was suitable for shouldering a high-frequency response, and V_A1_, V_A2_ and V_A3_ were suitable for shouldering a low-frequency response. V_A1_, V_A2_ and V_A3_ were larger than V_P_ at the low-frequency band, which could be attributed to a thicker ZnO layer with a small *C_E_*. The peak time of the maximum temperature variation rate increased when the thickness of the aerosol ZnO film increased due to the small temperature variation rate and large thermal capacity. V_T_ was the integrated voltage responsivity found by combining V_P_ with V_A1_, V_A2_ and V_A3_, which presented a compensatory effect to redeem the drawbacks of V_A1_, V_A2_ and V_A3_ with a tardy response at a high-frequency band and V_P_ with low voltage responsivity at a low-frequency band.

**Figure 10:**
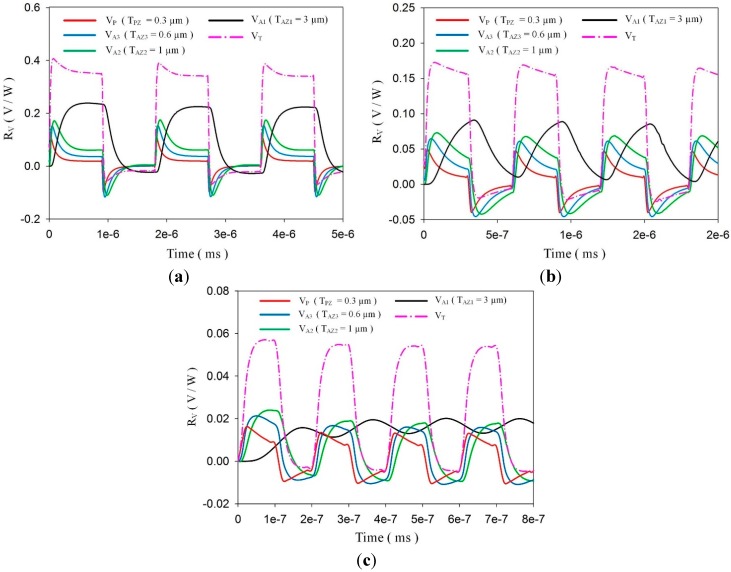
Calculated Voltage responsivities of a meliorated multi-frequency band pyroelectric sensor with T_PZ_ = 0.3 μm, T_AZ1_ = 3 μm, T_AZ2_ = 1 μm and T_AZ3_ = 0.6 μm under incident irradiation power modulated by various chopping frequencies of about (**a**) 14 KHz, (**b**) 33 KHz and (**c**) 100 KHz.

Transient temperature fields with various chopping frequencies were simulated for further calculation of voltage responsivities when the meliorated multi-frequency band pyroelectric sensors were exposed to irradiation power with various chopping periods. A square waveform was produced using a programmable function generator to modulate the irradiation power with chopping frequencies of about 14 KHz, 33 KHz and 100 KHz. Figure 10 shows the voltage responsivities of a meliorated multi-frequency band pyroelectric sensor with T_PZ_ = 0.3 μm, T_AZ1_ = 3 μm, T_AZ2_ = 1 μm and T_AZ3_ = 0.6 μm when the incident irradiation power was modulated with various chopping frequencies of about 14 KHz, 33 KHz and 100 KHz. The shape of the integrated voltage responsivity of V_T_ at three chopping frequencies almost approached a square wave. Furthermore, the amplitude of the integrated voltage responsivity of V_T_ increased when chopping frequencies decreased. This trend conformed to Equation (3). Moreover, the heat absorption of pyroelectric elements decreased for further transforming into electrical energy while the chopping frequency increased. Therefore, V_T_ generated by gradually increasing the thicknesses of the ZnO layers had a compensatory effect among the ZnO layers with various thicknesses. It was obvious that V_T_ generated by several ZnO layers with various thicknesses would be advantageous in expanding the sensing frequency band with a rapid response. The wave form of V_T_ approached a square shape, which presented that V_T_ possessed identical voltage responsivity to the rapid response. This implied that the integrated voltage responsivity of V_T_ generated by meliorated multi-frequency band pyroelectric sensors could present performances of various pyroelectric devices at various sensing frequency bands.

**Figure 11:**
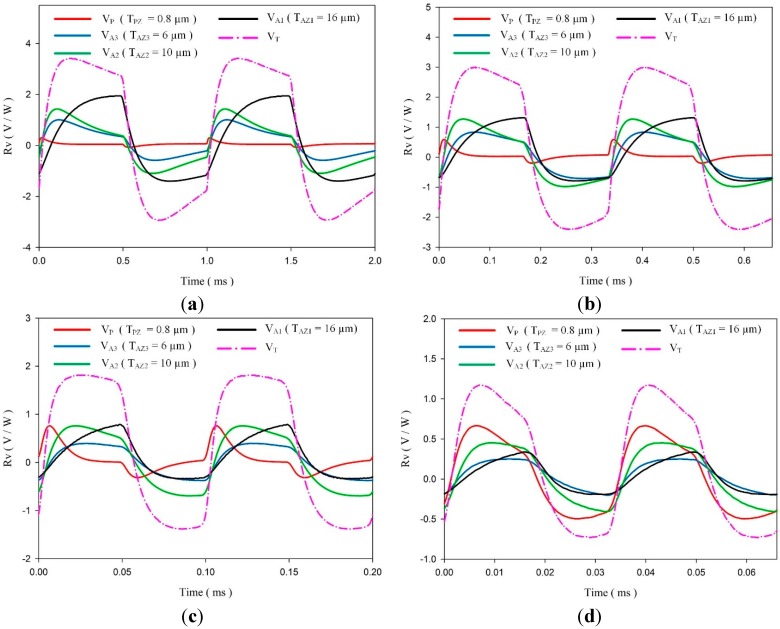
Measured Voltage responsivities of the meliorated multi-frequency band pyroelectric sensor with T_PZ_ = 0.8 μm, T_AZ3_ = 6 μm, T_AZ2_ = 10 μm and T_AZ1_ = 16 μm under an incident irradiation power modulated by various chopping frequencies of about (**a**) 1 KHz, (**b**) 3 KHz, (**c**) 10 KHz and (**d**) 30 KHz.

An experimental setup was used to verify the above analytical results. The IR laser beam was chopped and molded as a square wave with various modulated frequencies (1 KHz, 3 KHz, 10 KHz and 30 KHz) to obtain temperature variation rates in the meliorated multi-frequency band pyroelectric device by a programmable function generator. Figure 11 shows the voltage responsivities of the fabricated multi-frequency band pyroelectric device with T_PZ_ = 0.8 μm, T_AZ3_ = 6 μm, T_AZ2_ = 10 μm and T_AZ1_ = 16 μm when the incident irradiation power was modulated by various chopping frequencies of about 1 KHz, 3 KHz, 10 KHz and 30 KHz. The amplitude of the integrated voltage responsivity of V_T_ increased while chopping frequencies decreased due to the increasing period to absorb thermal energy. V_T_ presented almost a square form. This indicated that the response time was hardly affected by using various thicknesses of ZnO layers at these frequencies. Moreover, the intensity of V_T_ was almost identical when using various thicknesses of ZnO layers at a fixed frequency. The fabricated multi-frequency band pyroelectric device at a modulated frequency of 1 KHz presented a low-frequency property. The intensity of V_T_ increased, and it did not possess a tardy response. Furthermore, the fabricated multi-frequency band pyroelectric device at a modulated frequency of 10 KHz presented a high-frequency property. Although the intensity of V_T_ decreased, the sensing frequency band increased. This implied that the voltage responsivity could hold on a peak value with a larger sensing frequency band. However, a higher frequency of 30 KHz was over the multi-frequency band range of the fabricated device. Although the intensity of V_T_ was improved, the sensing frequency bands of V_T_ and V_P_ were the same due to the weaknesses of V_A1_, V_A2_ and V_A3_ at higher frequencies. Therefore, the fabricated multi-frequency band pyroelectric device was effective in the range of 1 KHz~10 KHz with a rapid response and large voltage responsivity. Hence, the multi-frequency band pyroelectric device could enhance pyroelectric sensors detecting subjects with various speeds or frequencies without dependence on the thicknesses of the pyroelectric elements.

## 4. Conclusions

A general pyroelectric sensor with a single pyroelectric layer only has a single sensing frequency band. However, multi-frequency sensing tasks could improve applications of pyroelectric sensors for detecting subjects with various speeds or frequencies under a fine responsivity. The combination of a thinner sputtered ZnO layer and three thicker aerosol ZnO layers was proved useful in the present design. The tactic of gradually increasing the thickness of the ZnO layers presented a compensatory effect for redeeming drawbacks among the ZnO layers. The fabricated device was effective within the range of 1 KHz~10 KHz with a rapid response and large voltage responsivity while ZnO layers with four thicknesses of about 0.8 μm, 6 μm, 10 μm and 16 μm were used for fabricating a meliorated multi-frequency band pyroelectric sensor. A meliorated multi-frequency band pyroelectric sensor was successfully designed, analyzed, and fabricated in the present study.
